# Metabolic Adaptations in Cancer Stem Cells

**DOI:** 10.3389/fonc.2020.01010

**Published:** 2020-06-25

**Authors:** Umesh Prasad Yadav, Tashvinder Singh, Pramit Kumar, Praveen Sharma, Harsimrat Kaur, Sadhana Sharma, Sandeep Singh, Santosh Kumar, Kapil Mehta

**Affiliations:** ^1^Laboratory of Molecular Medicine, Department of Human Genetics and Molecular Medicine, Central University of Punjab, Bathinda, India; ^2^Department of Biochemistry, All India Institute of Medical Sciences, Patna, India; ^3^Desh Bhagat Dental College, Mandi Gobindgarh, India; ^4^Department of Experimental Therapeutics, MD Anderson Cancer Centre, The University of Texas, Houston, TX, United States

**Keywords:** cancer stem cell, self-renewal, metabolism, oxidative phosphorylation, anaerobic respiration

## Abstract

Cancer stem cells (CSCs) are a small and elusive subpopulation of self-renewing cancer cells with remarkable ability to initiate, propagate, and spread the malignant disease. In addition, they exhibit increased resistance to anticancer therapies, thereby contributing to disease relapse. CSCs are reported to be present in many tumor types such as melanoma, sarcoma, mammary tumors, colon cancer and other solid tumors. These cells from different tumors show unique energetic and metabolic pathways. For example, CSCs from one type of tumor may predominantly use aerobic glycolysis, while from another tumor type may utilize oxidative phosphorylation. Most commonly these cells use fatty acid oxidation and ketone bodies as the main source of energy production. CSCs have a remarkable ability to reprogram their metabolism in order to survive under adverse conditions such as hypoxia, acidosis, and starvation. There is increasing interest to identify molecular targets that can be utilized to kill CSCs and to control their growth. In this review, we discuss how an understanding of the unique metabolism of CSCs from different tumors can offer promising strategies for targeting CSCs and hence to prevent disease relapse and to treat the metastatic disease.

## Introduction

Cancer is the result of the accumulation of genetic and epigenetic changes that eventually lead to uncontrolled cell growth and the gain of invasive functions by cancer cells. Cancer cells from different tumors can exhibit different properties ranging from low to high metastatic potential, low to high cellular plasticity, and chemosensitivity to chemoresistance. Cancerous mass is itself extremely heterogeneous in terms of metabolism, proliferating ability, and morphology due to genetic and epigenetic variations in intra-tumor subpopulations (1). Cancer stem cells (CSCs) represent a small subpopulation of cancer cells within these heterogeneous tumors that are aggressive, undifferentiated, with self-renewal ability, sensitivity to ROS molecules, and are known for hyperactive metabolism. CSCs were first identified in AML in 1997 as a rare and phenotypically different subset of tumor cells. These cells are able to divide in immuno-compromised mice and to give rise to leukemic progenitor cells and then to differentiated tumor cells (2). It is now well-known that altered glucose (through aerobic glycolysis known as “Warburg effect”) and lipid metabolism (β-oxidation) is a characteristic feature of CSCs, deciding the fate of their progression and self-renewal. This altered metabolism is now considered an important hallmark of CSCs and targeting cancer metabolism is emerging as a crucial therapy (3–5). Pioneer work by the German physiologist, Otto Warburg, revealed that metabolic processes are exploited to meet increased energy demands for proliferation and survival. The highly proliferative cancerous state usually differs from normal metabolism by using high glucose uptake in the presence of oxygen (through glycolysis) producing biomass and lactate rather than relying on oxidative phosphorylation for energy (3). Cancer cells increase their glucose consumption with an equal rate of increased glucose supply, while glutamine utilization is also increased for macromolecule synthesis. Altered glucose metabolism by cancer cells is critical for their growth, and to respond to the environmental changes (6).

## Oncometabolism

**Figure 1:**
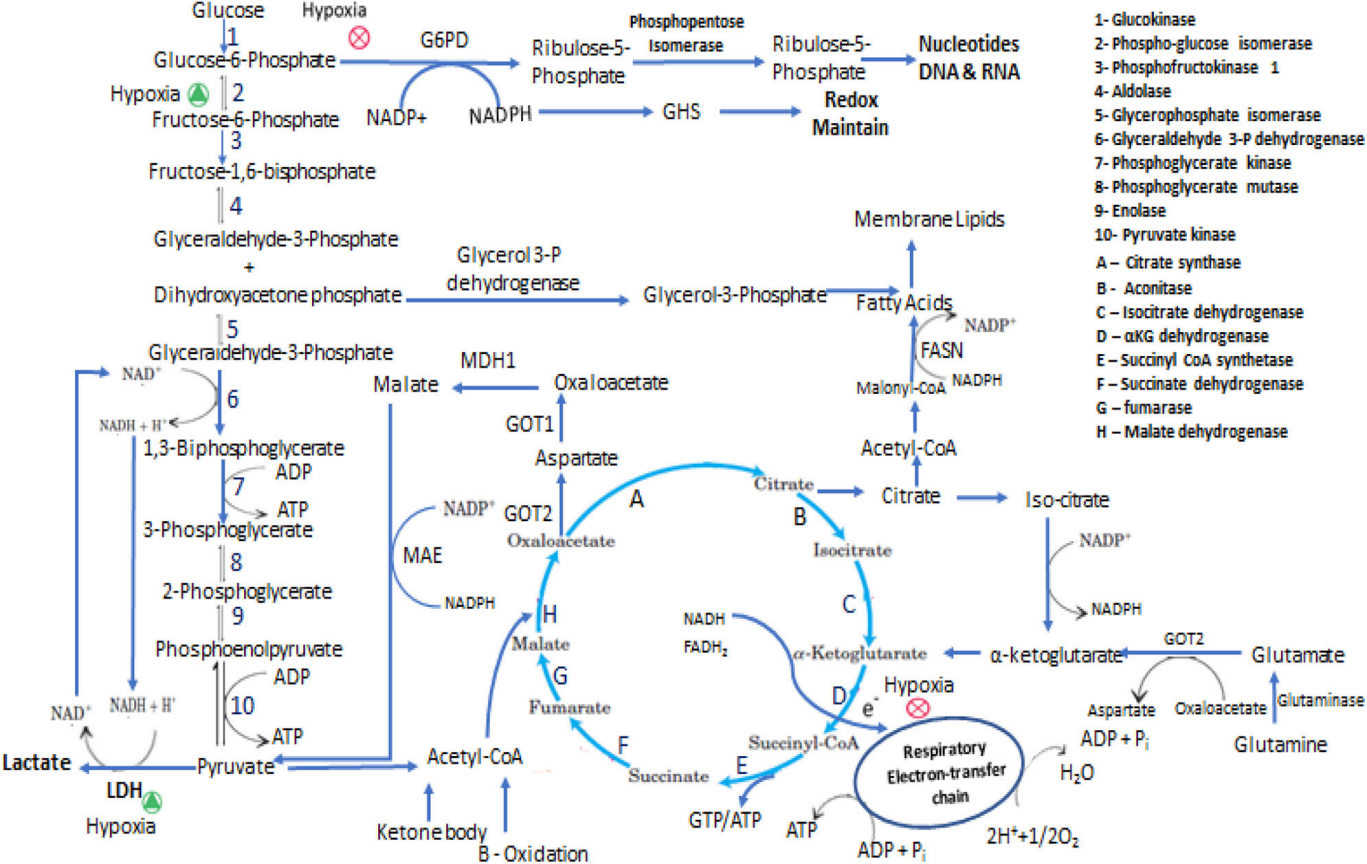
Representation of some of the metabolic adaptation and reprogramming of cancer stem cells. In the interconnected metabolic pathways of glucose, the key junctions are glycolysis and pentose phosphate pathway and central molecules are Glucose-6-phosphate, Pyruvate and Acetyl-CoA. In proliferative state cells metabolized Glucose mainly through PPP and perform less glycolysis and ATP has been produced from TCA cycle via respiratory electron-transfer chain by Acetyl-CoA coming either from pyruvate, Fatty acids and Amino acids oxidation. In Hypoxic condition PPP is inhibited and glucose metabolized through anaerobic glycolytic pathway. In presence of low glucose intermediate metabolites and ATP are produced mainly by glutaminolysis and fatty acids oxidation. CSCs synthesize fatty acids for membrane formation and oxidized this for energy production the equilibrium between anabolism and catabolism of fatty acids is maintain by NADPH and ATP requirement.

Metabolic rewiring is essential to meet the increased energy demands by cancer cells for their survival under stressful conditions and to generate metabolic intermediates to meet their rapid growth demands. These metabolic alterations could be the reason for higher proliferation, aggressive invasiveness, and chemo-resistant tumor cells (7–9). The importance of metabolic reprogramming was highlighted in a study showing that disallowing altered metabolic homeostasis using negative modulator of glucose metabolism, slowed the metabolic growth of triple-negative breast cancer (Negative for estrogen, progesterone, and epidermal growth factor 2 receptor) cells due to lowering of lactate production (9). Normal cells predominantly utilize glucose through glycolysis followed by subsequent metabolism of pyruvate via the tricarboxylic acid (TCA) cycle and oxidative phosphorylation (OxPhos) in mitochondria. But highly proliferative cancer cells need to adapt to cellular metabolism to provide regular support for the increased proliferation rate and rapidly generating higher amounts of ATP than non-proliferative cells. The normal proliferating cells such as regenerating hepatocyte or proliferative cells in culture media also utilize glucose anaerobically (10). The metabolic features of the proliferative cell either of normal or transformed differs from non-proliferative cells (11). The normal tissues cells switch their cellular metabolism for proliferation from OxPhos to aerobic glycolysis and once differentiated revert back to OxPhos. Cellular signaling helps in cell proliferation and in maintaining the undifferentiated state of cancer cells as well as in restructuring the metabolism during cancer cell proliferation. This high energy production metabolism is essential to fulfill the energy demands, maintain the increased demand for macromolecules, and tight regulation of the cellular redox status (12). Cancer cells meet their energy demand by metabolizing glucose to lactate via glycolysis in the presence of oxygen rather than mitochondrial OxPhos (13). The Warburg effect i.e., aerobic glycolysis is primarily found in malignant tumor cells for maintenance and survival for cancer cells due to impairment of mitochondrial function in the cancer cells. Studies show that in many cancers, mitochondria are functional and still the cells utilize aerobic glycolysis and show reverse Warburg effect when glycolysis is inhibited (14). The metabolic alterations of a cancer cell depend on various factors such as a change in oncogenes, tumor suppression genes, hypoxic microenvironment, mtDNA mutation, genetic factors, proliferation rate, and others (15). The reason for the decreased mitochondrial OxPhos could be deleted copies of mitochondrial pyruvate carrier (MPC) or its decreased expression. Indeed, forced expression of MPC-1 and MPC-2 increased the pyruvate oxidation (11, 16). Also, the glycolytic rate of ATP production is faster than OxPhos, but the energetic yield of glycolysis of 2 ATP is considerably lower in comparison to OxPhos, which produces 32 ATPs per molecule of glucose (17). For ATP generation, besides glycolysis, other important pathways involved are fatty acid oxidation and amino acid catabolism as shown in Figure 1.

Glycolysis, TCA, and OxPhos are an integral component of biosynthesis pathways that are needed to produce metabolic intermediates e.g., acetyl-CoA serves as a substrate for both catabolic and anabolic processes (18). Cells metabolism varies according to cell type i.e., adipocytes undergo increased lipolysis to provide simple lipid for β-oxidation in tumor cells. Adipocytes play a supportive role in tumor progression and metastasis by providing nutrients (lipids in this case) and adipokines that facilitate tumor growth by metabolizing adipocytes (19). Other studies suggest the polymorphism of mitochondria among benign, low malignant and malignant ovarian tumor. For example, platinum-resistant sublines such as SKOW3/CDPP, SKOW3/CBP, A2780/CDDP, and A2780/CBP have lower expression of electron transport chain proteins such as ATP- α, PRDX3, PHB, ETF and ALDH as compared to platinum-sensitive cells SKOW3 and A2780 (20, 21). Lactic acid generated during glycolysis elevates NF-kB mediated IL-8 expression and enhances the tumor progression and angiogenesis (22). Chemokine such as IL-8 mediates tumor progression, angiogenesis, and metastasis in both omental adipocytes and endothelial cells (19, 22). Acidic pH is another important factor regulating enzymatic microenvironment of tumor cells. It is observed that acidic pH results in a significant increase in metastasis of both weak and strong melanoma cell lines A375P and C8161, respectively (23). Lactic acid produced in the cell decreases the pH level activating the MMPs and Cathepsin B, and subsequently increased degradation of collagen IV, enhancing matrix degradation and tumor invasion thus supporting the acidification mediated invasion hypothesis both *in*-*vitro* and *in*-*vivo* (24). The Warburg effect i.e., aerobic glycolysis is primarily found in malignant tumor cells in the presence of oxygen while some cancerous cells acquire glycolytic metabolic phenotype only because of the hypoxic environment (25). Besides the overwhelming described role of lactate in tumor energy metabolism (hyperactive glycolysis mostly due to hypoxic environment), the role of oxidative phosphorylation is still important for fulfilling energy demands, macromolecule biosynthesis in tumor cells (15, 26, 27). Now it is feasible to validate the inevitable role of different energy metabolic processes and their metabolic intermediates participating in macromolecule biosynthesis, cell survival, and supporting metastatic properties. Targeting CSC metabolism thus represents a promising approach to halt tumor growth and disease relapse by understanding their biology and designing novel therapeutic modalities (4, 28).

## Heterogeneity of CSCs

Cancer is not a single disease but a group of diseases in which cells share some common features of abnormal cellular processes with extremely heterogeneous metabolic features in each type of cancer. Even within the same tumor, constituent cells are heterogeneous and metabolic phenotypes vary from one cell to another.Despite predominant aerobic glycolytic metabolism and elevated glycolytic enzymes, proliferating cancer cells have poor prognosis in various types of cancer (29). Some studies have reported both inter- and intratumor metabolic heterogeneity within the same type of tumors (30, 31). According to somatic mutation theory, cancer arises from somatic mutations in cells that undergo clonal selection followed by expansion and ultimately becoming malignant. All somatic mutations are not cancer drivers as most but some are passive. One study reported that the prevalence of the somatic mutation in a kinase gene in different types of tumors (lung, breast, colorectal, gastric, ovarian) does not show the mutation in 73 cases out of 210 cases (32). Somatic mutation analysis of NOTCH1, NOTCH2, NOTCH3, TP53, CDKN2A, and other genes by biopsy in normal eyelid epidermis exposed to ultraviolet light of four donors indicated that these driving mutations help in a positive selection over normal tissue for development of colonies which are non-malignant and non- invasive. These genes are often expressed in squamous cell carcinoma (SCC) and are mutated in other skin cancers also. The clones are genetically heterogeneous and the driver mutations transform cells into malignant phenotype. Although the CDKN2A gene is not associated with positive selection over normal tissue but has a positive impact on progression to advanced-stage disease. Similarly, many somatic mutations found in normal esophageal epithelium tissue could yield heterogeneous colonies that could lead to esophageal SCC in presence of driver mutation. The RNA sequencing of 29 normal tissues out of 6,700 tissue samples revealed multiple somatic variants (33). Studies conducted using deep genome sequencing, histopathological studies or molecular marker analyses revealed a surprising morphological, genetic and clinical heterogeneity of cancer cells that fluctuates within the tumor mass (34–36). There are two theories that explain the reason for heterogeneity; clonal variation and cancer stem cell theory; both vary with tumor subtypes. Clonal variations theory supports the role for genetic, epigenetic, and micro-environment changes that contribute to tumor heterogeneity where tumor cells differ in phenotypic and metabolic processes (37, 38). Whereas, the cancer stem cells theory supports the notion that transformed stem cells (sub populated part of the tumor mass) acquire the properties like high tumorigenic and malignant potential to generate differentiated tumor cell pools (39). The cancer therapeutics should be developed on the basis of tumor type and evaluating CSCs to identify the origin and reason for the problem that will help to find the solution (40). The tumor cells are phenotypically and functionally heterogeneous and this heterogeneity could be intra-tumor or inter-tumor. One such study conducted on 72 patients using FDG-PET analysis has quantified the intratumoral metabolic heterogeneity in primary cervix tumors indicating its relevance with tumor volume rather than tumor stage or histology (41). A similar study conducted on 93 patients with metabolic heterogeneity in cervical cancer using FDG-PET evaluated the significance of heterogeneity as a prognostic marker for predicting tumor relapse. Higher heterogeneity was seen in patients with poor survival and high disease relapse rates than in non-relapsed cancer patients (42). The CSCs contribute to this tumor heterogeneity within a single tumor mass and is responsible for reestablishing the same phenotypic heterogeneity (as in parent tumor) elsewhere upon serial passaging or transplanting *in-vivo* (43). Improved technology has uncovered additional features of heterogeneity of tumors including variations in cell surface markers, tumor growth, and response to therapy. There is strong evidence supporting that multiple tumor cell subpopulations exist within single cancer e.g., single subcutaneous tumor contained 6 clonal variations from slow-growing to fast-growing melanomas (44), karyotypic heterogeneity in fibrosarcoma (45), and genetic heterogeneity in mouse mammary tumor (46). Similarly, the tumor cell heterogeneity was reported in colon cancer (47) and other transplantable and inter-convertible solid tumors (48, 49). The heterogeneous tumors generally comprised of undifferentiated CSCs, supportive cells, differentiated tumor progenitors, and tumor-infiltrating cells (2, 50). In addition, revertible phenotypic heterogeneity in melanoma cells from the patient's sample without hierarchy (51) and cells contributing to variable tumorigenic frequency in SCID mice from single melanoma tumor has been observed (52). Furthermore, human glioma contains subpopulations of cells with morphological and karyotypic heterogeneity that further gets intensified upon clonal variation and microenvironment. These subpopulations of human glioma cells differed in morphological (fibrous, squamous, and astrocytes), chemosensitivity, and rate of tumor growth (53–55). While most of the studies point out the relationship between variations of phenotype and metabolic process, such is not always the case. Phenotypic heterogeneity of CSCs/clonal variation-derived differentiated tumorsare not necessarilyinvasive. These genes are often expressed in SCC and mutated in other skin cancersalso. The clones are genetically heterogenous and further driver mutation transform the cell into malignant. CDKN2A gene is not associated with positive selection over normal tissue but on advanced stage has positive selection impact. Similar observations found that mosaicism of somatic mutation found in physiological normal esophageal epithelium generating heterogenous colonies and driver mutations leading toesophageal SCC. Isolated tumor cells from metastatic colon cancer show self-renewal as well as tumor-initiating properties and maintain stemness up to several generations, resulting in homogeneity of tumor subpopulation (56). Within a tumor mass, microenvironmental stress-mediated cancer cell's response can result in heterogeneous gene expression; where some cells are more sensitive to these stresses than others. The malignant efficiency of heterogeneous tumor cells differs in terms of tumor initiation, invasiveness, metastasis, and chemo-resistance (57). The variable nature of cancer cells includes some highly active and self-renewal aggressive cells (referred to as CSCs) that are modulated by epigenetic factors such as DNA and histone modifications (DNA methylation, histone trimethylation, and mono acetylation), and chromatin modifiers (58, 59). CSCs represent a small and elusive subpopulation of cancer cells within a tumor mass with stem cell properties and are responsible for enhanced tumorigenesis, EMT-mediated metastasis, relapse, resistance to combinatorial treatment and variant epigenetic expression (60–63). CSCs divide to replenish the tumor cells pool in a symmetrical manner whereas asymmetrical cell division gives rise to non-CSCs that are less tumorigenic, less proliferative, poorly metastatic, and more differentiated. These differentiated non-CSCs tumor cells exhibit non-heterogeneous nature of the tumor where the majority of cells in the primary tumor do not show stemness properties. According to recent evidence, these CSCs and non-CSCs may exhibit inter-convertible plasticity (1, 64). The heterogeneity of CSCs and plasticity in differentiated tumor cells (non-CSCs) supports the inter-convertible feature of CSCs and heterogeneous tumor mass (64)On the basis of phenotypic and genetic heterogeneity, tumor cells are arranged in hierarchical order with the CSCs comes at the top while the most differentiated tumor cells at the bottom (65). Multiple CSCs have also been reported in many other cancers including prostate, lung, liver, pancreas, kidney, bladder, ovary, and brain etc. (66). Isolated pure normal human breast “stem cells” showed same marker expression as of breast tumors and more CSCs were isolated from undifferentiated grade 3 tumors than the differentiated grade 1 tumor. Taken together these observations suggest that CSCs give rise to more heterogeneous tumors than non-CSCs cancer cells (67). In ovarian cancer too, spheroid cells are known to have CSCs like properties with increased ALDH activity and show high tumorigenic and metastatic potential both *in-vitro* and *in-vivo* and are more resistant to cisplatin (68). Recent studies documented that most of the solid breast tumor cell lines expressing CD44 (basal-like cells), CD24 (luminal-like cells), PROCR, and ESA are highly tumorigenic and show EMT markers on their surface and play role metastasis, instead of commonly thought CD44+ /CD24–/low and ALDH expression. The studies also advocated that not only CSC marker signature in breast cancer cells is heterogeneous but many subsets of CSC exist that vary from patient to patient and may be related to the individual genetic makeup of the tumor (69).

## CSC Markers and Circulating Cancer Stem Cells

CSCs from different cancer types can be isolated based on the presence of cell surface markers. CSCs express stemness markers such as CD133, CD34, CD24, CD44, CD166, and EpCAM, ESA, ALDH1on cell surfaces (66, 70). BCNU resistant subpopulation of Glioma cells exhibits stem cell-like properties, and express CD133, CD117, CD90, CD71, and CD45, also revealing tumorigenicity *in vivo* upon transfer to SCID mice (71). The markers expressed by these CSCs are required for their stemness, invasiveness, and tumorigenic properties and differ in different subtypes of cancers. For example, CD44+/CD24- in breast CSCs (72), ALDH1+ breast carcinoma (73); CD44 expression in prostate CSCs (74); CD133 (75), ALDH1(76), and CD44 (77) expression in lung CSCs; ALDH1expressing epithelial CSCs (78); while human glioblastoma expressing SSEA-1 (79), EGFR (80), CD44 and Id1 (81). These cancer stem cell markers are essential for their self -renewal, migratory ability, and tumorigenesis such as in the case of the role of CD133 and CXCR4 in pancreatic CSCs (82). The CXCR4 is inevitably responsible for tumor cell invasion to the site of metastasis and tumor cell mobility (pseudopodia formation and actin rearrangement) (83, 84). Brain tumor CSCs expressing CD133 antigen were able to develop tumors *in-vivo* in SCID mice while the CD133-negative brain tumor cells failed to establish tumors in these mice (85). All these findings suggest that CSCs exhibit particular kinds of stemness markers responsible for self-renewal and tumorigenicity. The therapeutic intervention targeting CSCs markers is still an undiscovered field of study. It not only could delay the cancer progression but may possibly kill CSCs having tumorigenic/stemness properties. There is a special circulating subset of CSCs known as circulating cancer stem cells (CCSCs). The CCSCs detected and analyzed by Raman imaging from four breast cancer subtypes, showed the expression of CD133 marker. Upon culture of CCSCs in breast cancer differential media these cells showed changes in the expression of cell surface markers such as Her2 and EGFR (beside CD133) suggesting their differentiation into Her2+ breast cancer. The CCSCs were endowed with self-renewal ability, tumorigenicity, differentiation, stemness, and metastatic property both *in-vitro* and *in-vivo* (86). Isolating CCSCs from a heterogeneous CSCs population from advanced-stage tumors may offer new insight into cancer metastasis and disease relapse. Poor prognosis and relapse with chemo-resistivity were reported in cervical cancer having chromosomal aberration and high genetic heterogeneity that generally developed during cancer progression (87). As in the case of cervical CSCs, traditional anticancer therapies do not work due to overexpression of drug efflux transporter i.e., ABCG2 in undifferentiated tumor subpopulation indicating its role in maintaining stemness (88). Liver metastatic colorectal cancer cells express high levels of various progenitor markers such as EpCAM, CD44, CD24, and CEA-CAM along with CDX1. Cells expressing these markers show a close correlation with stemness, disease progression, and susceptibility to chemotherapy however their continuous exposure to chemotherapy resulted in the development of drug resistance phenotype. These observations suggest that CSCs mediated heterogeneity of tumors is important for metastasis and chemoresistance and that failure to target these cells results in tumor relapse.

## EMT and CSCs

The epithelial-to-mesenchymal transition (EMT) defines the transition of some tumor cells from differentiated to an undifferentiated state, linking it with cancer progression and metastasis. EMT-related transcription factors such as Wnt, TGF-β, Notch ligands regulate EMT mediated cancer progression, tumor cell plasticity and increased tumorigenicity (89, 90). Thus, EMT is an important factor regulating heterogeneity, metastatic ability, and plasticity of the tumor cells. Plasticity in breast CSCs can be decoded with interchangeable expression of two marker profiles i.e., CD24-CD44+ (mesenchymal type) and slightly higher tumorigenic potent ALDH+ (epithelial type). Breast cancer cells undergoing EMT show the stemness and tumorigenic properties which are regulated by the tumor microenvironment (91). EMT pathway is multi-step process, which starts with loss of epithelial markers and acquiring the mesenchymal characteristicsenabling them to metastasizetodistantorgansvia activation of proteases, degradation of extracellular matrix and formation of new blood vessels (angiogenesis). EMT is a fundamental first step in successful invasion of cancer cells from primary tumor to distant organs in response to sensing the low nutrient supplies at primary tumor site. *In vitro* induction of EMT in human mammary epithelial cells is associated with loss of epithelial marker proteins, gain of mesenchymal marker proteins, ability to form spheroids, anchorage independent growth and acquisition of stem cell-like phenotype (92, 93). Similar*ex*-*vivo* results were reported in samples isolated from human breast carcinoma and normal mouse mammary stem cells with CD44^high^/CD24^low^ expression (94). Human mammary epithelial cells induced simultaneously by Ras-MAPK and EMT pathway activation exhibited both tumorigenic and stemness properties. Combined ectopic expression of H-Ras^v12^ and TGF-β1 shortened the time taken to acquire mesenchymal phenotype in CD24+ cells (95). Activation of the Notch pathway resulted in increased NF-kB signaling and upregulated the expression of mesenchymal markers as observed in gemcitabine-resistant pancreatic cancer cells. Conversely, inhibition of the notch pathway or its downstream targets attenuated NF-kB activation, invasion, loss of epithelial markers and gain of mesenchymal markers in gemcitabine-resistant pancreatic cancer cells (96). Upregulated expression of EMT markers in hepatocellular carcinoma (HCC) is considered to be essential for their malignant phenotype as well as for transformation of non-differentiated malignant cell (97). Various transcription activators/repressors and miRNAs either stabilize or antagonize the EMT-induced stem cell traits and undifferentiated state in cancer cells. These miRNAs silence the protein expression of mRNA transcripts by guiding them for degradation while transcriptional repressors and activators downregulate or upregulate the gene transcriptional activity, respectively. ZEB-1 a transcriptional repressor of epithelial marker genes (e.g., E-cadherin) is an inducer of EMT and inhibits the expression of miR-200c and miR-141, the negative regulators of an EMT pathway. Thus, ZEB1 and ZEB2 are responsible for upregulating and downregulating the mesenchymal and epithelial phenotypes, respectively, while miR-200 family has just the opposite effect (98–102). Expression of miR-200 family genes is inhibited when cancer cells acquire the stem cell traits and its expression inhibits mammosphere formation. However, the transient downregulation of miR-200b had no observable effect on CSCs formation. MiR-200b inhibits the Suz12 and its regulated E-cadherin repression (103). Micro RNAs that inhibit stemness (such as miR-200a, miR-200b, miR-200c, miR-141, miR-429, miR-205, miR-203 and miR-183) do so by inhibiting the stem cell regulators such as ZEB1, ZEB2, SIP1, Sox2, Klf4 and hence repress the stemness maintenance and EMT induced CSCs formation (104, 105). EMT regulation is not restricted to the miRNA repression/activation or to the action of activators/repressors such as ZEB1, TGF-β1 and Ras-MAPK pathway; rather it is controlled by many transcriptional factors. Wnt/β-catenin signaling is a key positive regulator of EMT and CSCs formation. Indeed, the high Wnt/β-catenin signaling correlates with the progression of EMT, increased malignant phenotype and acquiring of stem cell traits (106–111). Disrupted Wnt signaling regulates the expression of β-catenin in colorectal cancer cells and is another example of transcriptional activators of oncogenes. Overexpressed β-catenin in cells undergoing EMT at the invasive front was observed in colorectal cancer while such expression was absent in normal colon epithelial cells present far from the invasion site (106). High canonical Wnt/β-catenin signaling in breast cancer demonstrates the action of β-catenin-TCF complex-mediated Snail1 activity and thus regulating EMT in Axin2 dependent pathway (107). PGE2 mediated C-terminal phosphorylation of β-catenin stabilizes it and upregulates Wnt signaling which is the conserved path for hematopoietic stem cell (HSC) formation, self-renewal, and for their sustenance (110). In yet another study, the prevalence of β-catenin expression in T-cell malignancy and non-Hodgkin lymphomas were analyzed and the activated Wnt signaling and highly accumulated β-catenin levels were observed in the nucleus in almost one-third of the tumor samples with some having gained the functional mutation in the β-catenin gene (109). HSCs with β-catenin deletion have difficulty in maintaining the prolonged growth and stemness ability without having any effect on differentiation to their subsequent lineage. *In* v*ivo* transplantation of cells with β-catenin deletion and expressing BCR-ABL transcripts imparted hind limb paralysis but could not induce leukemic phenotype in mice, while cells expressing wild type β-catenin could successfully induce CML in mice. The loss of β-catenin attenuated the progression of CML and self-renewal ability of CML stem cells, thus β-catenin could be one of the few hallmarks of CSCs survival and tumorigenicity (108). Take together, these observations suggest that Wnt/β-catenin signaling has an important role in the development of tumorigenicity and stemness features of CSCs. Thus, we can conclude that EMT is a necessarily parallel path by multi-grades tumor cells or CSCs for cancer progression, and thus to acquire metastatic and stem cell-like properties.

## Metabolic Rewiring of Cancer Stem Cells

Energy metabolism is an important physiological function to support the survival of cells and is critical for cancer progression. Hepatocellular CSCs expressing CD133+ stemness marker showed increased glucose metabolism than CD133- cells while inhibiting glycolytic enzymes in CD133+ CSCs by siRNA reduced the expression of Sox2, Oct4, and Nanog genes important for stemness. Providing extracellular glucose to CD133- cells, increased their stemness property (112). Pancreatic CSCs expressing stemness markers exhibit its dependency on the non-canonical pathway of glutamine metabolism and displayed increased apoptosis and ROS- generation in response to glutamine deprivation from the cell cultures. The pancreatic CSCs were readily sensitized to radiotherapy, accumulated ROS- in response to inhibition of glutamine metabolism both *in-vitro* and *in-vivo* (113). In another study, pancreatic CSCs showed increased expression of pluripotent stem cell markers (CD133, SSEA-1, CD44, and CXCR4) in response to activation of Nodal/Activin signaling or expression of its downstream mediators. The Nodal/Activin signaling pathway is important for stemness properties as their inhibition resulted in the sensitization of pancreatic CSCs to gemcitabine and abrogated theirs *in vivo* tumorigenic potential (114). CSCs exhibit unique metabolic adaptation to physiological and metabolic stresses such as decreased energy source, hypoxia, pH of the microenvironment, etc., thus CSCs differ in metabolic processes when compared to the non-CSC differentiated tumor. Evidence reports the similarity between CSCs and normal stem cells in their ability to differentiate into more mature cells and to self-renewal. CSCs metabolism is predominantly glycolytic but depending on the tumor type can partly be dependent on OxPhos too (115). The preference of glycolytic metabolism in CSCs over OxPhos will not be totally correct as growing evidence suggests that OxPhos has an enormous effect on CSCs survivability (116). In one such study, it was suggested that glioblastoma CSCs rely on Imp2 regulated OxPhos for their energy demand, survivability, *in vitro* clonogenicity and tumorigenic properties. Depletion of the Imp2 impaired the OxPhos and subsequently resulted in the loss of stem cell properties in glioblastoma CSCs (117). Leukemia CSCs expressing CD34+/CD38- in a dormant state are characterized by low ROS- production and lower glycolysis dependency while Bcl-2 regulated OxPhos is the main source of energy. Inhibition of the Bcl-2 impaired the OxPhos pathway and subsequently eradicated the quiescent leukemia CSCs (118). There is metabolic difference between CSCs and normal stem cells and also between CSCs and differentiated tumor cells, so further studies are warranted to elucidate the role of preferred energy metabolic process over other metabolic processes in a particular type of CSCs (119). It has been reported in glioma cancer stem cell and progenitor cells that CSCs exhibit low glucose consumption, low lactate production, high ATP generation, and high mitochondrial oxidation for their energy demands than their differentiated glioma cell, suggesting OxPhos be the main source for metabolic and energy dependency. The factors that make the difference in metabolism include oxygen consumption rates, extracellular acidification rate, intracellular ATP level, glucose uptake, lactate production, pyruvate kinase M1 (PKM1) levels, and pyruvate kinase M2 (PKM2) expression, cell cycle duration (120). There are convincing reports demonstrating that pancreatic and lung CSCs too depends on OxPhos as the main source of energy production and cell survivability. High mitochondrial membrane potential, low mitochondrial DNA, low ATP and ROS-, lower oxygen consumption over glucose metabolism were reported in lung CSCs when compared to non-lung CSCs. Thus, mitochondrial metabolism is a significant measure of differentiating lung CSCs from non-lung CSCs and may be used to signify other CSCs types (121). Pancreatic CSCs with low metabolic plasticity are highly dependent on OxPhos and are sensitized to metformin drug when compared to primarily glycolytic insensitive non-CSCs (122). However, other investigators have reported that CSCs are more dependent on anaerobic glucose metabolism. The metabolism not only differs in differentiated and CSCs but also varies from their progeny. Breast CSCs predominantly use glycolytic metabolism with a high level of LDH-1 and PKM2 (anaerobic glycolytic enzymes) and low β-oxidation as its main metabolic feature; anaerobic glycolysis inhibitor, 2-deoxyglucose reduced the cell growth and survivability of breast CSCs (123). Human U87 glioblastoma CSCs isolated from xenograft modeled mice exhibited dependency on glycolytic metabolism along with low OxPhos and preference for hypoxia-mediated stemness and chemoresistance. *In vitro* inhibition of glycolysis induced cytotoxicity and retarded the tumor growth in mice (124). PKM2 plays an important role in anaerobic glycolysis, though is not essential for cancer cell survival or progression and is known to inhibit aerobic glycolysis (125, 126). However, in CSCs, which are more like non-dividing cancer cells, CSCs may require PKM2 for energy related functions as well as maintaining their status (126, 127).

Osteosarcoma CSCs are dependent on high glycolysis and low OxPhos for energy and survival when compared to non-CSCs MG63 cells. LDHA inhibition using sodium oxamate resulted in greater cytotoxicity against CSCs than the MG63 cells while low glucose induced increased fragmented mitochondrial morphology in contrast to network mitochondrial morphology in MG63 cells. These findings support the inability of CSCs mitochondria to become more active under glucose starvation (128). Breast CSCs showed expression of high glycolytic and low OxPhos related enzymes and fewer mitochondria when compared to non-tumorigenic cancer cells indicative of hyper glycolytic metabolism in these CSCs. DCA mediated increase in PDH enzyme (involved in OxPhos) expression in CSCs resulted in increased cytotoxicity *in vitro* and decreased tumor growth *in vivo* (129). Hypoxia-induced stemness and glycolytic dependency of breast CSCs is the main reason for chemoresistance in CSCs. However, glycolytic inhibitor along with combinatorial drugs under these conditions makes the CSCs more sensitive to conventional therapies. Leukemia CSCs exhibiting low FAO and high Myc expression with increased concentration of lactate, citrate, and succinate could be positively linked with CD133+ stemness marker as compared to non-cancer stem cells. High lactic acid production is correlated with hyperactive glycolysis in CD133+ cells, through the enzymatic activity of LDHA (112, 130). Radioresistant nasopharyngeal carcinoma CSCs and hypoxia resistant spheroid cells of ovarian cancer exhibiting stemness properties mainly follows the glycolytic pathway and use the byproduct amino acids in biosynthetic pathways rather than their complete oxidation and FA biosynthesis, respectively (68, 131). While the mitochondrial respiration was shut off in nasopharyngeal carcinoma CSCs, the mitochondrial activity and biogenesis were still active in expressing low-ROS and higher TFAM, POLG, and PGC-1a genes (131). The microenvironment of the cells gives clues about active metabolic programming. For example, Paneth cells with increased glycolytic metabolism that supports intestinal stem cells are known to be Lgr+ crypt base columnar cells (CBCs). Paneth cells are functional in the intestinal crypt and produce lactate which is converted into pyruvate to support mitochondrial OxPhos in CBCs having high mitochondrial activity and low redox burden (132). In general, stem cells such as hematopoietic stem cells and human mesenchymal stem cells mainly follow glycolytic metabolism to fulfill their energy demands and shift to other metabolic pathways upon their differentiation. Osteogenic induction of human mesenchymal stem cells is followed by increased mt-DNA copy number, OxPhos, and decrease in cytosolic ROS-, glycolytic metabolism, lactic acid formation while the accumulation of ROS- and increased OxPhos mitigates osteogenic differentiation of human mesenchymal stem cells (133). Glycolytic dependent and hypoxia-resistant HSCs showed low mitochondrial potential, low OxPhos, and high stemness properties and increased expression of HIF-1α both *in vitro* and *in vivo* (134). These findings suggest that CSCs can exhibit variable metabolic features depending on their origin and the microenvironment in which they metastasize. Metastatic competent 4T1 breast cancer cells expressed increased capacity for both glycolytic and oxidative metabolism compared to non-metastatic 67NR cells. For example, changes such as PDK-1 support glycolytic metabolism in liver cells but OxPhos metabolism in lung and bone cells (135). Hyperoxia mediated aerobic glycolysis downregulates ROS- in CSCs while in the absence of glucose and hypoxic conditions CSCs metabolism shifts to the mitochondrial respiration. These variations in glucose metabolisms may be influenced by the tumor microenvironment (136).

## Glycolysis and Oxphos Correlation in Tumorigenicity

Many metabolic processes e.g., Glycolysis and OxPhos (primary), fatty acid oxidation (secondary) that are regulated by the microenvironment may vary in ATP production in order to fulfill the energy demands and to fuel the anabolic pathways for cancer cells growth (137). The glycolytic product pyruvate undergoes OxPhos under normal aerobic conditions but is converted to lactate by lactate dehydrogenase-A (LDH-A) under an anaerobic state and is transported to the extracellular fluid through MCT (subfamily of cell membrane transporters). These pathways are co-regulated to maintain energy balance. While glycolysis provides instant energy and is the main source for ATP under hypoxic environment, the energetic yield via ATP production is too low compared to ATP production via OxPhos pathway (15). Glycolysis is predominantly activated in tumor cells and aerobic glycolytic cells under hypoxic conditions but OxPhos too does not lose its ability to generate energy (ATP) required for tumor growth. OxPhos is rather suppressed by enhanced glycolysis. Suppression of one metabolic pathway in tumor cells is paved away by another metabolic process to be activated, and it is dependent on the tumor microenvironment. Tumor cells might have functional OxPhos (137–141) despite having multiple mtDNA mutations (142) due to large number of heterogenous genomic copies. The stroma adjacent to tumor cells exhibits high glycolysis which acts as a fuel for active OxPhos in epithelial tumor cells with functional mitochondria (143). Overall, factors such as nutrients, oxygen availability, tumor microenvironment, cell's energy demands, etc., determine the switch between different metabolic pathways.

## Metabolism of Glucose in CSCS

As previously stated, glucose is one of the main sources of energy for both CSCs and differentiated tumor cells, providing instant energy but with a lower yield. Glycolysis serves two purposes, one it provides energy readily to the cell, and second, the glycolytic end-product (pyruvate) is involved in the biosynthesis of amino acids (through TCA cycle intermediates), and lipids (precursor for acetyl CoA). Thus, meeting the need of proliferating metastatic cancer cells, which require high energy and precursors for macromolecule biosynthesis in a very short time frame (3, 144). Increased glucose concentration and higher expression of glucose transporters such as GLUT and SGLT, increase glycolysis and is linked with increased viability of tumor cells and CSCs (145–147). There are two classes of glucose transporters which facilitate the transport of glucose by different mechanism.

The GLUT transporters facilitate glucose uptake along the gradient while sodium-dependent SGLT transporters does against the gradient by (148–150). GLUT1 is primarily responsible for glucose uptake in tumor cells e.g., relieving glucose depleted prostate tumor cells from oxidative stress (151, 152). There are also some reports supporting the role of GLUT3 in glucose uptake for cancer progression e.g., in non-small cell lung metastasis and colorectal cancer (153, 154). The dependency of CSCs for high glycolytic and low OxPhos metabolism increases glucose uptake induced by glucose's own concentration. Reduced glucose concentration, genetic knockdown or pharmacological inhibition of GLUT1 attenuated the stemness properties and spheroid formation in pancreatic, ovarian, and glioblastoma CSCs without compromising the cell viability (155). Similarly, the reduced tumorigenic potential was observed when pancreatic CSCs were treated with WZB117 prior to their administration in immuno-compromised mice (155). High glucose concentration resulted in increased GLUT1 and GLUT3 mRNA expression that was associated with increased HIF-1α expression (156). Pentose phosphate pathway (PPP) and glycolysis are inter-connected to each other through glucose-6P, pyruvate, and acetyl CoA. Glucose-6P is derived from glucose by the catalytic action of hexokinase, the enzyme that represents the starting link between glycolysis and PPP (157). Glucose metabolism through PPP pathway is necessary for HIF-1α stabilization in glucose-dependent hypoxic tumor cells and subsequently supports angiogenesis (156). Glioma CSCs undergoing hypoxia show up-regulated mRNA expression for both the glycolytic and PPP genes but protein expression was limited to only glycolytic enzymes such as LDH-A and hexokinase-2, while downregulation of PPP enzymes such as glucose-6-phosphate dehydrogenase, 6-phosphogluconate dehydrogenase and transketolase like protein was observed. Acute hypoxia although upregulated migration ability but slowed down proliferation activity, whereas acute oxygenation had just the opposite effect i.e., decreased migration. Under both the conditions increase in apoptotic cells was observed. Rapidly dividing cells have an up-regulated PPP pathway whereas under acute hypoxic conditions pathway shifted toward glycolysis (158). The glycolytic byproduct, lactate is a potent metabolite for inducing angiogenesis and invasiveness in macrophages and vascularisation of endothelial cells (159). The MCT1 mediated lactate uptake increases HIF-1α expression in endothelial cells while blocking MCT1 reduces the VEGFR2 and HIF-1α expression in both Genetic knockdown of glycolytic and PPP enzymes showed the same metabolic correlation both *in vitro* and *in vivo* (160). Glucose metabolism and lactate production were seen in at half of the contact inhibited mouse neural progenitor cells while low glucose/glutamine stimulated the proliferation in fibroblastic cells. Equal PPP metabolic activity among contact inhibited and proliferating cells led to the flux of ribose phosphate into glycolysis and nucleotide biosynthesis, respectively. Inhibition of PPP pathway, on the other hand, induced more apoptotic effect in contact inhibited cells than fibroblastic cells (161). Thus the profound activity of glycolysis in a hypoxic environment makes it an essential metabolic process for tumor cells residing in the center of the tumor mass with the lower blood supply of oxygen and nutrients.HUVEC and BAEC cells (162). Proliferating and migrating glioblastoma cells differ in their metabolic nature of consuming glucose and its downstream pathways as seen in *in* v*itro* and *in vivo* studies. Migratory cells showed increased and decreased expression of glycolytic enzymes and PPP enzymes, respectively. In contrast, proliferating cells had higher PPP enzymes expression but low glycolysis metabolic expression.

## Metabolism of Lipids in CSCS

Fatty acid synthase (FASN) is an intracellular enzyme that is involved in fatty acid synthesis by converting malonyl-CoA and acetyl-CoA into palmitate in three steps using NADPH. Domain I of FASN catalyzes the formation of carbon-carbon bond between malonyl-CoA and acetyl-CoA and subsequent reduction of the elongated fatty acid chain by domain II followed by thioesterase activity of domain III (163). CSCs play an important role in cancer progression and are often reported to have increased activity of FASN (164). These elongated FA chains are structural components of membranes and their *de novo* synthesis is highly activated in tumor cells compared to normal tissues (165, 166). There are reports supporting increased FASN activity and expression in various human cancers such as breast cancer (167–169), thyroid cancer (170), ovarian cancer (171), prostate cancer (172, 173), oral cancer (174–176), colorectal cancer (177), endometrial metastatic cancer (178) and in mesothelioma (179), renal cancer (180), and retinoblastoma (181, 182). All these studies, inevitably describe FASN as an important metabolic target in cancer therapeutics. Increased FASN expression plays a fundamental role in maintaining the stemness, invasiveness and tumor-forming abilities of Glioma CSCs. Inhibition of FASN in these cells reduced their invasiveness and spheroid forming ability along with the reduced expression of stemness markers such as CD133, FABP7, while increased expression of differentiation marker GFAP (183). NANOG, a transcriptional factor is responsible for promoting the stemness properties and it is preferentially relevant to the mitochondrial metabolism. It reduces the expression of mitochondrial OxPhos genes and elevates the expression of mitochondrial fatty acid oxidation genes in tumor-initiating cells/CSCs of hepatocellular carcinoma.Inhibition of OxPhos gene COX6A2 or COX15 reduced ROS- production whereas activation of the fatty acid oxidation (FAO) gene inhibited glucose utilization via OxPhos and also altered the anaplerotic reactions that help in maintaining the stemness of cells. Induction of the Oxphos gene and inhibition of FAO reduced the spheroid formation ability of cells (184). Pancreatic CSCs showed increased expression of glycolytic and PPP enzymes and immensely induced FASN expression while decreased TCA metabolic enzymes in comparison to pancreatic non-CSCs. Pharmacological inhibition through cerulenin and atorvastatin reduced the cell-viability and dramatically distorted the mesenchymal appearance and spheroid forming ability of pancreatic CSCs (185). Lipid droplets store the excess fatty acids in the cell and act as a reservoir to maintain cholesterol levels, triglyceride levels for cell membranes synthesis, and conserving the energy. Lipid droplets are the key regulators of CSCs metabolism besides their role in the storage and synthesis of inflammatory factors (186, 187). FASN hyperactivation in colorectal cancer cells leads to lipid droplets accumulation, increased FAO and reliability on aerobic glycolysis, suggesting the dependence of tumor cells on stored lipid forms for energy homeostasis (188). All these studies, unequivocally suggest that FASN is an important metabolic target in cancer therapeutics. Stearoyl-CoA-desaturase-1(SCD1) is an enzyme involved in fatty acid synthesis by converting saturated fatty acids into mono-unsaturated FA which serves as a substrate for other lipids and has conserved activity in brain and pancreas (189). SCD1 expression is elevated in CSCs and serves as a marker for poor prognosis of lung adenocarcinoma along with other stemness markers. Pharmacological inhibition of SCD1 resulted in increased susceptibility of lung CSCs to cisplatin-induced apoptosis in (190). Besides aerobic glycolysis and TCA cycle, the role of FAO in satisfying energy demands, chemo- and immune-resistance, stemness, and cancer progression could be demonstrated by analyzing the lipolytic phenotype of CSCs that ispredominantly dictated by the tumor microenvironment (191). Gonadal adipose tissue enriched in pro-inflammatory leukemic CSCs undergoes lipolysis to serve as repository of lipids needed by the leukemic CSCs to maintain stemness and survival. Leukemic-CSCs with increased FAO and upregulated CD36 (FA transporter) expression exhibit chemoresistance while *in vivo* and *ex vivo* studies with CD36 knockout cells showed decreased tumor size when injected in mice. These findings suggest the importance of CD36 along with FAO for phenotypic and metabolic distinction in comparison to non-leukemic CSCs (192). FAO has been reported to be a crucial metabolic pathway for breast CSCs as pharmacological inhibition of FAO, reduced the cell viability, ATP levels, and tumor-forming ability in breast CSCs. While JAK-STAT is the key signal transduction pathway responsible for FAO-mediated chemoresistance and tumorigenicity as inhibition of STAT3 reduced the β-oxidation in these CSCs (193). CSCs display high levels of carnitine palmitoyltransferase (CPT) which facilitates the transport of fatty acid from the cytosol to mitochondria for their oxidation (194). In CSCs, CPT1 serves as an anti-apoptotic molecule by interacting with BH3 family of proteins and acts as a cell survival factor (194, 195). This is interesting how CSCs simultaneously complete synthesis and oxidation of fatty acids. During energy depleted state, NADPH generation by the PPP is impaired and this results in decreased FA synthesis. Under these conditions mitochondrial generation of NADPH is important. FAO provides acetyl-CoA which enters into the TCA cycle to form citrate and malate the substrates for NADPH producing enzymes isocitrate dehydrogenase and malic, respectively. AMPK mediated increased β-oxidation and decreased fatty acid synthesis during energy stress maintains the NADPH levels in tumor cells, thus supporting their survivability (196). Cancer cells optimize their requirements for growth and proliferation by regulating the lipid anabolic and catabolic switch. NADPH and ATP demand play an important role to equilibrate the fatty acid synthesis and oxidation in cancer cells.

## Mitochondria and Stemness in CSCS

Mitochondrion is the cell organelle involved in the generation of ATP. In addition, they are also involved in many cellular processes such as maintaining the redox state, generation of ROS-, maintaining cytosolic Ca++ level, helping the biosynthesis, and inducing apoptotic death of the cell. Multiple reports have suggested that, despite enhanced glycolysis, cancer cells can produce a significant fraction of their ATP via mitochondrial respiration (197–199). Metabolic plasticity is a characteristic of cancer cells and anaerobic glycolysis performed by cancer cells is not only due to mitochondrial dysfunctions. Mitochondria are necessary for the development and progression of cancer as removal of cancer cell mitochondria diminished cancer growth rate and tumorigenicity. When mtDNA is depleted from tumor cells (ρ° cells) they show reduced growth rate, poor colony-forming ability and considerably reduced tumor growth in mice (200–202). These observations suggest that functional mitochondria are required for the successful survival of cancer cells. Mitochondrial DNA mutations have been reported in many types of cancers such as renal adenocarcinoma, colon cancer cell, head, and neck cancer, breast cancer, prostate and bladder cancer, ovarian cancer, thyroid cancer and neuroblastoma (201, 203–207). MtDNA mutations are a risk factor in some cancer cell populations and are a positive selection in tumorigenesis. Mitochondrial mutations and reduced mtDNA cause stress in mitochondria and thus mitochondria reprogram itself. Studies in breast cancer cell line MCF-7 and normal mammary epithelial cells MCF-10A revealed that mtDNA mutation or reduced mtDNA induces the EMT and CSC phenotype resulting in increased migration and colonization at distant places. This contention was further supported by treatment of MCF-7 and MCF10A cells with ethidium bromide (50 ng/ml) up to 5 passages that resulted in an increased number and increased life span of mammospheres formed by reduced mtDNA.Moreover, flow cytometry analysis showed that cell surface stem cell markers (high CD44^+^ and low CD24^−^) are abundantly expressed in reduced mtDNA cells than normal and reverted cancer cells (201). Mutation or knockdown of mitochondrial transcription factor TFAM which is encoded by the nucleus and acts as the main transcription factor for mtDNA and also helps in mtDNA replication and packaging mitigated the tumorigenic potential of cancer cells (208). The mitochondrial morphology, its distribution, and mtDNA status in CSC vary when compared to the differentiated cancer cells or their normal counterparts. Other studies performed on lung CSCs A549 having high expression of CD34, CD133, c-kit, Twist1, Sox2, Oct4, NANOG, and Bmi show reduced mtDNA and higher Δψm in comparison to non-lung CSC. CSCs have high Δψm which suggest that the mitochondria play a role in cell differentiation, tumorigenesis and maintaining the stemness of cell (121). Mutations in oncogenes and mtDNA initiate the formation of cancer/CSC by altering the wave of transcription, which is overblown by the hypoxia and microenvironments, and the mitochondrial stress results in transcription to reprogram the cancer cell metabolism. Decreased mtDNA and mutation coordinates with a nucleus and reprograms the metabolism by sending mitochondrial retrograde signaling (208). Recent research supported that there might be a strong interplay between mitochondria and its role in stemness as mitochondria being an important player in providing energy for the maintenance of stemness. Overall it is necessary to explore the mitochondrial physiology in CSCs and its mechanism in promoting the therapy-resistance (209). While stemness induces suppression of mitochondrial biogenesis and maturity, it activates gene expression of glycolytic enzymes with increased substrate consumption and lactic acid production (210). It has been reported that CSCs from both ovarian adenocarcinoma and cervical SCC express stemness marker, low-ROS- levels, low lactate production, high amino acid intermediates of TCA when grown into spheroids (3D-culture) while opposite effects were seen in adherent tumor cells (2D-culture). These observations suggest that TCA and overall mitochondrial respiration guides and maintains the stemness properties of CSCs and their inhibition is activated upon differentiation, mediating the shifting of CSCs from OxPhos to glycolytic metabolism (211). Mitochondrial respiration and FAO are inter-linked where excess acetyl-CoA is used in TCA cycle. Ketones may too increase mitochondrial respiration and suppress ROS- production by its conversion into acetyl-CoA (212). The reduced ROS- level is favorable to CSCs survival under stress conditions and thus the utilization of ketone bodies may relieve CSCs from oxidative stress. While the ketone metabolism is active in the absence of nutrients, tumor cells and CSCs constitutively express it (213–217). Mitochondrial metabolic activity is also related to cell differentiation, as early passages of an adult primate stromal cell line have a higher OCR and a low ATP/mitochondrial DNA content compared with long-term cultured cells (218) but CD34+ hematopoietic stem cells have low mitochondrial OCR and mitochondrial mass (219). The HSC mitochondria play important roles in maintaining stemness and differentiation. However, whether the roles of CSC mitochondria are similar to HSC mitochondria or cancer cells, in general, is unknown. Two hypotheses on the origin of CSCs, both of which contribute to acute myeloid leukemia (220), have been proposed. One hypothesis supports that CSCs are derived from normal stem cells residing in various organs. Genetic mutations and epigenetic changes, which are crucial for the initiation and progression of tumor growth, accumulate in long-lived stem cells, and the transformation of stem cells into CSCs initiates the carcinogenesis. CSCs may also have a greater differentiation potential than normal stem cells. Another hypothesis assumes the existence of embryonic stem cell-like cells that transform into CSCs when they are exposed to damaging environmental factors. Additional differentiation and mutations of these cells may also contribute to the development of CSCs (221). As reported, ovarian CSCs show higher mitochondrial ROS- production and ΔΨm than non-CSCs. In addition, targeting mitochondrial biogenetics induced caspase-independent cell death in ovarian CSCs (222). In glioma CSCs, a higher mitochondrial reserve capacity was measured as compared to the differentiated cells (120). Glioblastoma CSCs also depend on OXPHOS for their energy production and survival (117). Besides, breast CSCs have higher ATP content compared to their differentiated progeny (223). Therefore, it is reasonable to believe that CSCs mitochondria show different roles and features depending on the cancer type and CSCs mitochondria differ from those of non-CSCs. Importantly, little information is available on the mitochondrial features related to energy metabolism and the ROS-/antioxidant enzyme system of CSCs in colon, stomach, liver, bone, and prostate cancer. Therefore, defining these features will be essential for developing a mitochondria-targeted therapeutic approach to facilitate the death of CSCs, and therefore, to reduce the risk of disease relapse and progression to refractory cancer.

Mitochondrial studies using whole-cell approaches make it difficult to distinguish mitochondria-specific effects from those contributed by the nucleus. This gap can be filled by using trans mitochondrial cybrid models in order to investigate the mitochondria-regulated energy and cancer pathways (224–226). Cybrid models using moderately metastatic triple-negative breast cancer cell line (SUM159) as a background control for nuclei and mitochondria and comparing them with the benign breast cells (MCF-10A or A1N4), and highly metastatic (MDA-MB-231) breast cancer cells can offer a good working model for this purpose. Indeed, these cybrids show the tumor-like properties both *in vitro* and *in vivo* according to their mitochondrial origin. Mitochondria obtained from benign cells almost completely abolished the tumorigenic properties of SUM159 cells when tested *in vitro* and *in vivo* (226). Proteomic and mass spectrometry analysis revealed several proteins related to mitochondrial. Fatty acid oxidation is also up regulated in cybrids with mitochondria derived from MDA 231 metastatic breast cancer cells. Knockdown of enzyme essential for fatty acid oxidation carnitine palmitoyltransferase-1(CPT1) or carnitine palmitoyltransferase-2 (CPT2) by shRNA significantly inhibited the migration potential and wound healing potential of these cells (226). Breast cancer patient dataset (n = 1,302) with long-term clinical follow-up showed that high CPT1A mRNA expression in tumors promotes distant metastasis (227). Altogether, these results show the role of mitochondrial energy reprogramming in fatty acid oxidation in CSCs and its significance in regulating the driving the protein of a major cancer pathway via its posttranslational modification.

## Metabolism of Glutamine in CSCs

Glutamine is a non-essential amino acid that plays a key role in energy and metabolic homeostasis during CSCs proliferation. It mediates these effects by regulating the consumption and uptake of other amino acids, hence maintaining mitochondrial redox potential and NADPH levels. Besides maintaining energy homeostasis, glutamine is the precursor of TCA cycle intermediates, nucleotides, proteins, and biosynthesis of other amino acids. CSCs from small cell lung carcinoma utilize glutamine actively in anaplerotic reaction in order to produce ATP for substrate-level phosphorylation. Complete inhibition of OxPhos and glycolysis by oligomycin and 2-DG in CSCs and non-CSCs caused more substrate-level phosphorylation in uPAR+ CSCs. Under respiratory distress conditions, substrate-level phosphorylation provides the necessary GTP/ATP to CSCs and this is more efficient under hypoxic conditions (228, 229). *In vivo* and *ex vivo* studies in OxPhos defective mtDNA mutated cybrid A6MT cells and wild type cells that have no mtDNA mutations, showed that Glutamine accelerates the proliferation of A6MT cell and its uptake was higher in mtDNA mutated cells than in wild type cells. Glutamine undergoes oxidative or reductive pathways depending on the severity of OxPhos defect. Isotopic C13-labeled glutamine supplemented in culture media for tracking the glutamine-glucose-αKG flux and metabolic fate of glutamine in A6MT cells suggested that glutamine enters the TCA cycle in the form αKG in a clockwise direction and provides GTP/ATP for substrate-level phosphorylation following its conversion into succinate as mentioned in Figure 1. Succinate is then converted into aspartate that migrates into the cytosol and subsequently converted into alanine and lactate. Published work from other laboratories have revealed similar results and suggest that Glutamine enters the TCA cycle and actively participates in anaplerotic reaction; the amount of lactate formed is less compared to that formed by glucose (230–232). These events are critical in order to maintain the pace of TCA cycle and to re-oxidize glycolytic NADH to generate NADPH which is a critical cofactor needed in reductive pathways such as during lipid synthesis as notified in Figure 1. Thus, glutamine has an important role in metabolic rewiring in OxPhos impaired cells to provide energy and other synthetics intermediates (233). Many researchers are attempting to target the glutamine-dependent self-renewal ability of CSCs as a novel therapeutic approach (234–236). For example, attempts to inhibit glutamine transamination in glioblastoma, blocking the increased glutamine regulated signaling pathways (mTOR pathway), or by targeting c-myc regulated glutamine uptake were shown to induce significant cytotoxicity to tumor cells (237, 238). Deamidation product of glutamine, along with cysteine and glycine, is an important component of glutathione that is needed to maintain the redox balance and NADP+/NADPH ratio (239). The dependency of cancer cells on glutamine metabolism for nitrogen and redox balance is correlated with their tumorigenic potential and tumor survival (240). mTOR is an important signaling pathway for glutamine metabolism and is differentially regulated by glutamine concentration of tumor cells. Colorectal CSCs grown in glutamine lacking media were more sensitive to metformin drug (relevant to mTOR pathway) while being resistant when media contained glutamine. Combinatorial treatment with metformin and glutaminase inhibitor, induced higher cytotoxic effect against colorectal CSCs than treatment with each drug individually. The colorectal CSCs had higher expression of glutamine transporter protein (ASCT2) than the non-CSCs cancer cells. Accordingly, the ASCT2 knockdown dramatically reduced the number of CD133+/CD44+ CSCs (241). In pancreatic CSCs, glutamine deficiency significantly decreased the stemness, self-renewal, and increased the ROS- production resulting in increased the apoptotic death of these cells (242). The uptake of extracellular proteins in pancreatic adenocarcinoma and bladder carcinoma cells, expressing oncogenic Ras, this mediated through macro-pinocytosis and to make glutamine via lysosomal degradation of these proteins (243). The α-ketoglutarate, an intermediate of TCA cycle, when supplemented to cell cultures as an alternate to Glutamine, may alter the cellular requirements for glutamine metabolism in order to synthesize nucleotides. CD34+/CD38– hematopoietic stem cells expressing high ASCT2 levels and glutamine metabolism show commitment to erythroid niche whereas abrogating glutamine metabolism leads to myeloid differentiation *in*-*vitro*. On the other hand, *in*-*vivo* antagonistic metabolism of glutamine and glucose regulates the differentiation of HSC between myeloid and erythroid (244)Glutamine is an important component in synthesis of glutathione (GSH), a tripeptide that serves as an intracellular antioxidant scavenger of ROS- and also is involved in DNA repair, activation of transcription factors, cell cycle regulation, and calcium homeostasis (245). Glutamine metabolizing enzymes such as glutamine synthetase and glutamate-oxaloacetate transaminase (GOT1 and GOT2) are highly expressed in CSCs (113). Glutamine metabolism varies with cancer types andgenerally proceeds through two pathways; acting as a mediator of anaplerotic flux through TCA cycle intermediates and a precursor for nucleotide synthesis (246). Incorporation of glucose carbon in glutamine and TCA cycle intermediates, non-dependency of tumor cells on glutamine was seen in non-small-cell lung carcinoma cells *in*-*vivo*. This phenomenon wasregulated by changes in the microenvironment and indicated glucose as a carbon source. These observations suggested glucose mediated glutamine synthesis and TCA cycle metabolite replenishment with the help of pyruvate carboxylase rather than dependency on glutamine anaplerosis (247). Glioblastoma expressing aerobic glycolysis and active TCA cycle predominantly depended on glucose-mediated lipogenesis and on anaplerotic glutamine metabolism to regenerate TCA cycle intermediates for NADPH production. Generally, low reliability of TCA mediated metabolites and high preference for glutamine is seen in proliferating cells than in contact inhibited fibroblasts. These findings support the contention that contact inhibition may induce mild anapleroticresponse in favor to glutamine metabolism and strong anapleroticresponse in favorto pyruvate-oxaloacetate flux (161). Myc mediated glutamine catabolism to α-ketoglutarate, replenishes the reduced TCA cycle metabolite pool in response to hypoxic stress and low pyruvate availabilityfor TCA cycle. Generated α-ketoglutarate undergoes reverse TCA cycle to form citrate, which is exported to the cytosol to form oxaloacetate and Acetyl-CoA for lipid synthesis and redox homeostasis of cell (248). Glutamine metabolism is undoubtedly a major metabolic pathway in CSCs and could be a promising therapeutic target toblock its functions and aidingthe replenishment of metabolites for energy and redox homeostasis.

## Conclusion: CSCS Eradication by Targeting its Metabolism

It is important to understand the exact nature of factors in heterogeneous tumor mass that fuel the tumorigenic growth, drug refractoriness, and metastasis of less differentiated tumor cells to distant organs. It is also clear that CSCs play a major role in replenishing the tumor pool and as a source of differentiated tumor cells. The immortal nature of CSCs might be the reason why the tumors relapse even after most of the tumor mass is removed or eradicated (249). Oxphos and glycolysis remains the primary energy generation mechanisms for CSCs while metabolism of ktone bodies and fatty acids also contributed significantly. This may vary for each CSC type depending on the primary tumor from which these CSCs have developed. Yuan et al. has shown that multiple mutations in mtDNA may render compromised OxPhos function (142) thus pushing the cell toward glycylysis. On the other hand, several studies have shown that glycolytic tumors can not metabolize fatty acid and ketone bodies (230, 250, 251) for energy production. Individual mitochodnria are genetically heterogenous due to large copy number of the genome, each copy may have different mutations. This may overall produce almost all mitochondrial functional proteins, albeit in very low levels, thus enabling OxPhos to a smaller extent, thus explaining the partial contribution of energy by multiple mechanisms.

Metastatic and self-renewal property of CSCs (while being more quiescent) is tightly regulated by the mitochondrial respiration and glycolysis, while the ATP generation and fulfilling energy demandsare secondary events. While Myc suppression induced the sensitivity of CSCs to metformin, overexpression of Myc has just opposite effects making cancer cells behave like chemo-resistant pancreatic adenocarcinoma CSCs. Drugs targeting the OxPhos could be the primary aspect of therapeutic intervention in pancreatic CSCs as some drugs exclusively target the CSCs metabolism while showing no effect on non-CSCs (122). A rationale reasoning for metabolic immuned CSCs is still unknown where CSCs evade the immune response and may confuse the immune cells to distinguish itself from normal stem cells. Genomic analyses, mRNA profiling and mutational analysis of breast cancer (252), ovarian cancer (253), lung cancer (254), glioblastoma cancer (255), prostate cancer (256), gastric cancer (257), B-cell lymphoma (258), acute lymphoid leukemia (259), metastatic cancers (260), and melanomas (261) has been performed using sophisticated techniques such as MSK-IMPACT, next-generation sequencing, and whole-genome sequencing. These studies have provided a detailed and perspective view of the diverse genetic and epigenetic nature of different mutated genes in different tumor types that may be involved in the regulation of metabolic processes and proliferating efficiency based on their origin and microenvironment in which they reside. Future efforts to elucidate the mechanisms responsible for genetic, epigenetic and micro environment-induced changes in CSCs that regulate tumor progression and chemoresistance might offer therapeutic opportunities for successful intervention to block the progression of cancer to untreatable disease. Alternative approaches should be taken, while focusing on similarities between different CSCs. For example, common metabolic features that dictate the tumorigenic potential and stemness of CSCs should be targeted. Targeting of metabolic wiring should be investigatedduring two transitional states; (a) from normal stem cells to cancer stem cells, and (b) from cancer stem cells todifferentiated tumor cells, rather than using chemo- and radiation therapiestokillthe whole tumor mass that results in major side effects. While we have no knowledge on the metabolic adaptations that take placeduring normal stem cell to CSCs transition, only a handful studies have been done to understand the transition of CSCs to differentiated tumor cells.

## Author Contributions

KM, SK, and SSi organized topics and contributed in writing the manuscript. UY, TS, PS, and PK collected the literature and contributing in write up. UY, TS, PS, SSh, and HK helped in revising the manuscript. UY and PS designed the figure. All authors contributed to the article and approved the submitted version.

## Conflict of Interest

The authors declare that the research was conducted in the absence of any commercial or financial relationships that could be construed as a potential conflict of interest.

## Abbreviations

AML: Acute myeloid leukemia
CAM: cell adhesion molecule
CBC: Crypt base columnar
CD: Cluster of differentiation
CPT: Carnitine palmitoyl transferase
CSC: Cancer Stem Cell
CCSC: Circulating cancer stem cells
DCA: Dichloroacetic acid
EMT: Epithelial mesenchymal transition
ESCC: Esophageal squamous cell carcinoma
FAO: Fatty acid oxidation
FANS: Fatty acid synthase
HSC: Hematopoietic stem cells
MPC: Mitochondria pyruvate carrier
OxPhos: Oxidative phosphorylation
PDH: Pyruvate dehydrogenase
PK: Pyruvate kinase
PPP: Pentose phosphate pathway
FDG-PET: Fluorodeoxyglucose-Positron emission tomography
SCC: Squamous cell carcinoma
SCD1: Stearoyl-CoA-desaturase-1
SGLT: Sodium-glucose linked transporter.
